# The Role of β-Glycosylated Wall Teichoic Acids in the Reduction of Vancomycin Susceptibility in Vancomycin-Intermediate Staphylococcus aureus

**DOI:** 10.1128/Spectrum.00528-21

**Published:** 2021-10-20

**Authors:** Michael Hort, Ute Bertsche, Senada Nozinovic, Alina Dietrich, Anne Sophie Schrötter, Laura Mildenberger, Katharina Axtmann, Anne Berscheid, Gabriele Bierbaum

**Affiliations:** a Institute of Medical Microbiology, Immunology and Parasitology, University Clinics of Bonn, Bonn, Germany; b Department of Infection Biology, University of Tuebingen, Tuebingen, Germany; c Department of Chemistry, University of Bonn, Bonn, Germany; Riverside University Health System-Medical Center, Loma Linda University

**Keywords:** vancomycin-intermediate *S. aureus*, wall teichoic acids, *tarS*, two-component systems, vraSR, autolysis, peptidoglycan

## Abstract

Staphylococcus aureus is an opportunistic pathogen that causes a wide range of infections. Due to the rapid evolution of antibiotic resistance that leads to treatment failure, it is important to understand the underlying mechanisms. Here, the cell wall structures of several laboratory vancomycin-intermediate S. aureus (VISA) strains were analyzed. Among the VISA strains were S. aureus VC40, which accumulated 79 mutations, including most importantly 2 exchanges in the histidine-kinase VraS, and developed full resistance against vancomycin (MIC, 64 μg/ml); a revertant S. aureus VC40R, which has an additional mutation in *vraR* (MIC, 4 μg/ml); and S. aureus VraS(VC40), in which the 2 *vraS* mutations were reconstituted into a susceptible background (MIC, 4 μg/ml). A ultraperformance liquid chromatography (UPLC) analysis showed that S. aureus VC40 had a significantly decreased cross-linking of the peptidoglycan. Both S. aureus VC40 and S. aureus VraS(VC40) displayed reduced autolysis and an altered autolysin profile in a zymogram. Most striking was the significant increase in d-alanine and *N-*acetyl-d-glucosamine (GlcNAc) substitution of the wall teichoic acids (WTAs) in S. aureus VC40. Nuclear magnetic resonance (NMR) analysis revealed that this strain had mostly β-glycosylated WTAs in contrast to the other strains, which showed only the α-glycosylation peak. Salt stress induced the incorporation of β-GlcNAc anomers and drastically increased the vancomycin MIC for S. aureus VC40R. In addition, β-glycosylated WTAs decreased the binding affinity of AtlA, the major autolysin of S. aureus, to the cell wall, compared with α-glycosylated WTAs. In conclusion, there is a novel connection between wall teichoic acids, autolysis, and vancomycin susceptibility in S. aureus.

**IMPORTANCE** Infections with methicillin-resistant Staphylococcus aureus are commonly treated with vancomycin. This antibiotic inhibits cell wall biosynthesis by binding to the cell wall building block lipid II. We set out to characterize the mechanisms leading to decreased vancomycin susceptibility in a laboratory-generated strain, S. aureus VC40. This strain has an altered cell wall architecture with a thick cell wall with low cross-linking, which provides decoy binding sites for vancomycin. The low cross-linking, necessary for this resistance mechanism, decreases the stability of the cell wall against lytic enzymes, which separate the daughter cells. Protection against these enzymes is provided by another cell wall polymer, the teichoic acids, which contain an unusually high substitution with sugars in the β-conformation. By experimentally increasing the proportion of β-*N-*acetyl-d-glucosamine in a closely related isolate through the induction of salt stress, we could show that the β-conformation of the sugars plays a vital role in the resistance of S. aureus VC40.

## INTRODUCTION

Methicillin-resistant Staphylococcus aureus (MRSA) is a dominant cause of nosocomial diseases around the world. In a study of nonhospitalized adults in Germany, up to 40% of participants were colonized with S. aureus and 0.7% with MRSA (1). Since the late 1980s, the glycopeptide antibiotic vancomycin has been the drug of choice against MRSA (2). However, the introduction of a new antibiotic for the treatment of infections often leads to the evolution of new resistance mechanisms. The first clinical S. aureus strain with reduced susceptibility to vancomycin was reported from Japan in 1996 (3).

According to CLSI standards, S. aureus is classified as vancomycin susceptible with a MIC of up to 2 μg/ml, as vancomycin intermediate with an MIC between 4 and 8 μg/ml, and as resistant with an MIC equal to or above 16 μg/ml. Vancomycin-intermediate S. aureus (VISA) does not contain a *van* operon but instead develops reduced susceptibility through the accumulation of mutations during treatment with vancomycin (4). Multiple genes and mutations have been implicated in generating VISA from susceptible parent strains. These mutations are found in regulatory two-component systems (TCSs) like the essential *walKR* operon, *vraSR*, and *graSR*, involved in autolysis and cell wall metabolism. They also affect genes coding for the RNA polymerase subunit *rpoB*, wall teichoic acid attachment proteins like *msrR*, or proteolytic proteins like *clpP* (5–10). In the vast majority of S. aureus strains, a single mutation is not sufficient to convert a susceptible strain into a VISA strain; however, a WalR(K208R) amino acid exchange found in a clinical isolate yielded an MIC of 4 μg/ml (5, 11). Common characteristics among VISA include (i) a thickened cell wall, which often exhibits an increase in free d-Ala-d-Ala residues, due to a lower level of cross-linking; (ii) a decreased virulence; and (iii) a reduced autolysis (12). The decreased cross-linking of the cell wall described in several VISA strains (13, 14) enables adsorption of vancomycin to the free d-Ala-d-Ala residues and thereby prevents binding of vancomycin to lipid II via the so-called “clogging effect” (15). Both cross-linking through penicillin-binding proteins (PBPs) and peptidoglycan (PGN) turnover are influenced by the wall teichoic acids (WTAs) in the cell wall. The absence of WTA biosynthesis prevents the localization of PBP4 and AtlA, the major autolysin, to the division septum and instead disperses the enzymes throughout the cell surface (16, 17).

Previously, a laboratory VISA strain, S. aureus VC40, was generated through serial passage of S. aureus RN4220Δ*mutS* in the presence of increasing concentrations of vancomycin (18). The strain reached a vancomycin MIC of 64 μg/ml in brain heart infusion (BHI), which meets the criteria for vancomycin-resistant S. aureus (VRSA). Since S. aureus VC40 does not contain a *van* operon, like all other isolates categorized as VRSA, it is still labeled as a VISA strain (6). Whole-genome sequencing revealed a total of 79 mutations in 75 gene loci. Most noteworthy were two amino acid exchanges in VraS(L114S and D242G) and one in WalK(I544M). To judge the effect of the VraS exchanges, both mutations were reconstituted into the S. aureus NCTC8325 background, which increased the MIC against vancomycin from 1 to 4 μg/ml in BHI and also reduced Triton X-100-induced autolysis to a level comparable to S. aureus VC40 (6). S. aureus NCTC8325 was chosen for the reconstitution because it is *agr* positive and in this regard similar to S. aureus VC40, which had repaired the mutation in *agr* present in S. aureus RN4220Δ*mutS*. In addition, S. aureus VC40 had an increased cell wall thickness (62.36 nm diameter) compared with its parent strain and the reconstituted VraS mutant (S. aureus RN4220Δ*mutS,* 16.97 nm; S. aureus NCTC8325, 16.71 nm; S. aureus VraS(VC40), 38.25 nm) (6). This study elucidates the link between reduced vancomycin susceptibility and the compositional changes in the cell wall structure of S. aureus with a focus on wall teichoic acids, especially the WTA glycosylation pattern. We demonstrate here that the cell wall of S. aureus VC40 shows a very low cross-linking and is stabilized against the activity of the autolytic enzymes by an altered glycosylation and a higher content of wall teichoic acids.

## RESULTS

### Characteristics of the revertant strain S. aureus VC40R.

During the work with S. aureus VC40, a spontaneous revertant strain, S. aureus VC40R, was isolated. Genomic sequencing showed that this strain harbors an additional mutation in *vraR* leading to an M54T exchange, which is located directly adjacent to the phosphorylation site (D55). The strain exhibited a drastically reduced vancomycin MIC of 4 μg/ml in BHI medium compared with 64 μg/ml of the parent strain S. aureus VC40. Transmission electron microscopy of S. aureus VC40R showed a cell wall thickness of 55.03 nm (S. aureus RN4220Δ*mutS*, 16.97 nm; S. aureus VC40, 62.36 nm).

### The mutated form of VraR in S. aureus VC40R cannot be phosphorylated *in vitro*.

**FIG 1:**
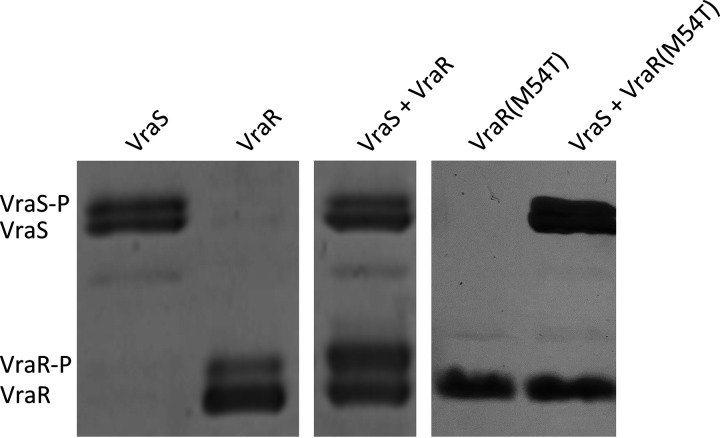
Phos-tag SDS-PAGE of *in vitro* phosphorylation of VraS, VraR, and VraR(M54T) after incubation with ATP. Phosphorylated bands (P) are indicated. The figure shows two representative gels. The presence of a seemingly phosphorylated protein band in the VraR control in the absence of VraS indicates either a partial phosphorylation of VraR by a kinase from E. coli or an impurity of the VraR protein preparation.

Strain S. aureus VC40 and its revertant strain S. aureus VC40R harbor two mutations in the kinase VraS, which leads to an increased expression of the VraRS regulon that responds to cell wall damage (6). Following a yet unknown signal, the histidine kinase VraS autophosphorylates and in turn transfers the phosphate group to the aspartate residue in position 55 in VraR. Then, this transcription factor undergoes a conformational change that promotes dimerization, which in turn leads to a higher affinity interaction between the regulator and cognate DNA sequences and alters target gene expression (19, 20). S. aureus VC40R contains an additional (M54T) mutation in the response regulator VraR. The phosphorylation of VraR(M54T) was assessed with the cognate kinase VraS via Phos-Tag SDS-PAGE (Fig. 1), which separates proteins by size and phosphorylation pattern. The Phos-Tag reagent interacts with phosphorylated proteins and slows their migration. In the SDS-PAGE, the VraR wild-type protein was phosphorylated by VraS. In contrast, the mutated VraR(M54T) did not show any phosphorylated band in the gel after incubation with VraS; hence, it lost the ability to be phosphorylated. This result was also confirmed by phosphorylation assays of VraR and VraR(M54T) in the presence of the phosphate donor lithium potassium acetyl phosphate as described by Tajbakhsh and Golemi-Kotra (20) (results not shown).

### The cell wall of S. aureus VC40 shows a very low cross-linking.

The PGN of S. aureus VC40 showed a significantly decreased amount of cross-linking in the ultraperformance liquid chromatography (UPLC) analysis compared with the control and with the other two VISA strains (Table 1). S. aureus VC40R and S. aureus VraS(VC40) did not significantly differ in their cross-linking compared with the respective susceptible control strains S. aureus RN4220Δ*mutS* and S. aureus NCTC8325. Consequently, S. aureus VC40 contained the largest amount of monomeric and dimeric PGN fragments, indicating a high concentration of free d-Ala-d-Ala residues. Since cross-linking of PGN is dependent on the transpeptidase activity of PBP4, the presence of this protein in the cell membrane was confirmed (see Fig. S1 in the supplemental material). The combination of an increased cell wall thickness and reduced cross-linking or an increased cell wall thickness alone should lead to an increased ability to absorb vancomycin to the PGN in the VISA strains S. aureus VC40, S. aureus VC40R, and S. aureus VraS(VC40). This ability was demonstrated in a vancomycin binding assay. While the vancomycin-susceptible S. aureus (VSSA) controls managed to bind only an estimated 5% of the added antibiotic, the S. aureus VC40 cells absorbed nearly 94% of vancomycin and the other two VISA strains showed intermediate binding (see Fig. S2 in the supplemental material).

**TABLE 1:** UPLC analysis of purified PGN

Strain	Crosslinking (%)	Monomers (%)	Dimers (%)	Rest (%)^*a*^
RN4220Δ*mutS*	71.39	10.25	14.73	75.02
VC40	62.89	14.26	21.69	64.05
VC40R	69.50	9.39	17.95	72.66
NCTC8325	69.83	11.17	16.44	72.39
VraS(VC40)	72.1	8.91	15.15	75.94

a Rest sums up all trimers, tetramers, and higher multimers.

### S. aureus VC40 shows a lower autolytic activity in a zymogram assay.

**FIG 2:**
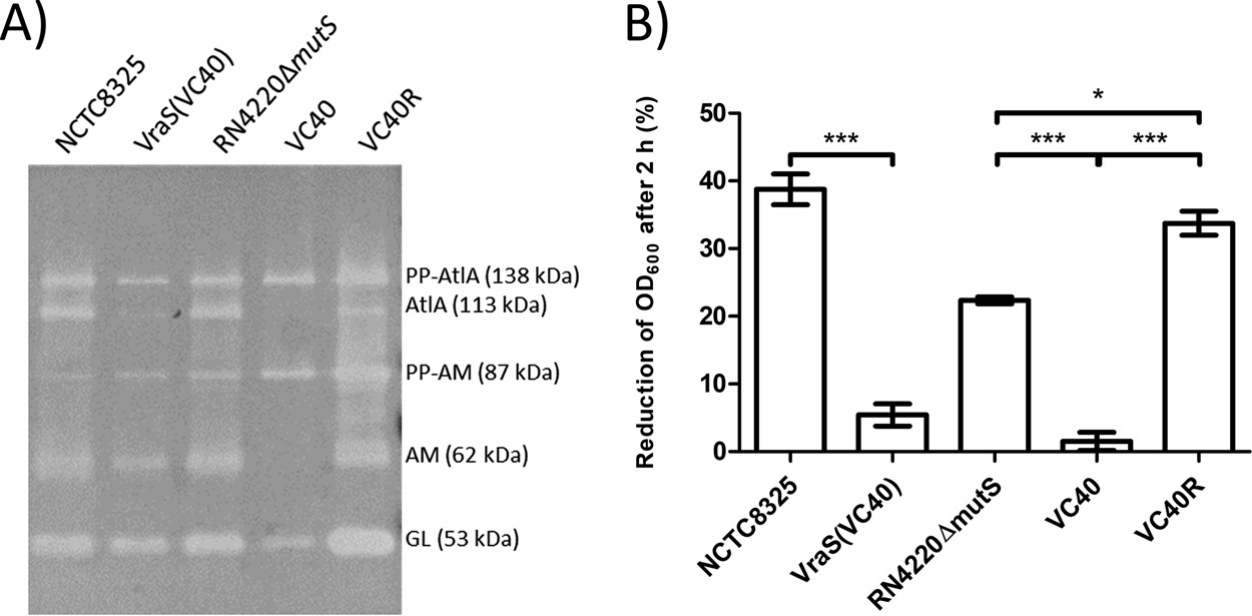
(A) Zymogram of isolated crude autolysin extract of all strains (volume adjusted to represent a similar amount of cells for all strains based on OD_600_) tested against M. luteus cells. Different fragments of the major autolysin AtlA are annotated (PP, propeptide; AM, amidase subunit with two repeat domains; GL, glucosaminidase subunit with one repeat domain). The figure shows a representative experiment; the zymogram was repeated three times. (B) Lysis assay measured after 2 hours of incubation with crude autolysin extract of each strain (volume adjusted to represent a similar amount of cells for all strains based on OD_600_) and purified PGN with WTA without d-alanine substitutions of each strain (*, *P* < 0.05; ***, *P* < 0.0005).

The extraction of crude autolysin revealed altered AtlA profiles in the zymogram (Fig. 2A). In contrast to all other strains, in S. aureus VC40, the amidase (AM) subunit was not visible, indicating that it either had been proteolytically processed or was not able to associate with the cell wall since the extracts had been prepared from washed cell pellets. The decreased concentration of the AtlA band in strain *S. aureus* VC40 was, to a lesser degree, also observed for S. aureus VraS(VC40). In contrast, all other strains, including the revertant strain S. aureus VC40R, showed comparable AtlA profiles in the zymogram. The altered AtlA profiles of S. aureus VC40 and S. aureus VraS(VC40) went hand in hand with the reduced lysis of purified, d-Ala-free PGN of these strains by their own crude autolysin extracts. Less than 10% of the PGN from S. aureus VC40 and S. aureus VraS(VC40) were lysed after an incubation for 2 h, demonstrating a significant difference compared with the other strains, which lysed up to 38% of the PGN (Fig. 2B).

### Wall teichoic acids inhibit autolysis in VISA.

**FIG 3:**
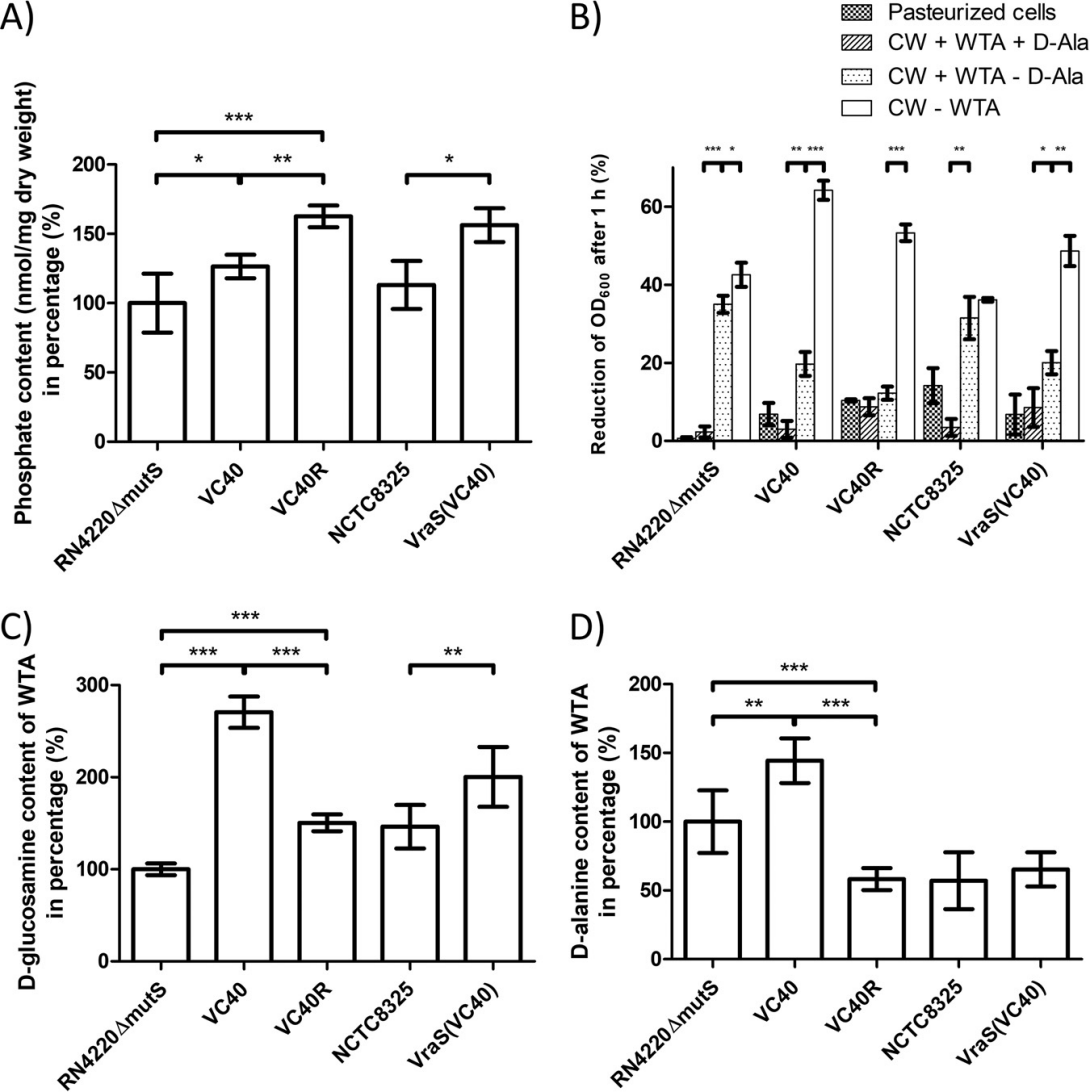
(A) Phosphate content of the isolated cell walls of different S. aureus strains, with S. aureus RN4220Δ*mutS* set to 100% (*, *P* < 0.05; **, *P* < 0.005; ***, *P* < 0.0005). (B) Lysis of cell wall fragments after different treatments, which were measured after 1 hour of incubation with 2-μg/ml AtlA. Namely, pasteurized whole cells, PGN with WTA and d-alanine substitutions, PGN with WTA after removal of d-alanine substitutions, and PGN without WTA were used as the substrates (*, *P* < 0.05; **, *P* < 0.005; ***, *P* < 0.0005). (C) *N*-acetyl-d-glucosamine substitution of the WTA based on sugar concentration in relation to the phosphate concentration with S. aureus RN4220Δ*mutS* set to 100% (**, *P* < 0.005; ***, *P* < 0.0005). (D) d-Alanine substitution of the WTA based on d-alanine concentration in relation to the phosphate concentration with S. aureus RN4220Δ*mutS* set to 100% (**, *P* < 0.005; ***, *P* < 0.0005).

The isolated cell walls, i.e., the peptidoglycan with teichoic acids still attached, of all VISA strains showed an increased phosphate concentration, which translates to an increased WTA content (Fig. 3A). This result was most pronounced for the revertant strain S. aureus VC40R with 62% more WTA. S. aureus VC40 and S. aureus VraS(VC40) increased the WTA concentration by 26% and 38% compared with their parent strains. Both PBP4 and AtlA activity are dependent on wall teichoic acids as temporal and spatial regulators (16) and a higher concentration of teichoic acids in VISA cells has been reported (21). In order to judge the effect of teichoic acids on the lysis characteristics of VISA cell walls, the lysis of different cell wall preparations was tested with purified AtlA. Removal of d-Ala substitutions from WTA, via incubation in 0.1 M NaOH, led to an increase in autolysis, which was more prominent for VSSA strains (Fig. 3B). Strikingly, in VISA strains, only the complete removal of WTA increased the lysis to an extent, which surpassed the lysis of the PGN isolated from vancomycin-susceptible strains. This effect was most notable in strain S. aureus VC40 and might be due to the low cross-linking of the peptidoglycan in this isolate.

### Wall teichoic acids are indispensable for vancomycin insensitivity in strain VC40.

The importance of teichoic acids for the resistance of VISA strains against vancomycin was further underlined by an observed synergy between vancomycin and tunicamycin in these strains (see Fig. S3 in the supplemental material). Tunicamycin is an antibiotic that prevents the first step of WTA biosynthesis, and it showed a synergistic effect with vancomycin for S. aureus VC40 (fractional inhibitory concentration [FIC] index, 0.125) and approached synergy for the revertant S. aureus VC40R (FIC index, 0.5). The vancomycin MIC of S. aureus VraS(V40) did decrease in the presence of tunicamycin, although the FIC index (0.75) did not indicate a synergism. In contrast, the control S. aureus RN4220Δ*mutS* showed no decrease in the vancomycin MIC with increasing tunicamycin concentrations (FIC index, 2).

### The wall teichoic acids of S. aureus VC40 contain significantly more *N*-acetyl-d-glucosamine and d-alanine.

Interestingly, S. aureus VC40 displayed an increased substitution of WTA with both *N*-acetyl-d-glucosamine (GlcNAc) and d-alanine (Fig. 3C and D). The S. aureus VC40 WTA accumulated over 2.7 times the amount of sugar and 1.4 times the amount of d-Ala compared with the parent strain. The other two VISA strains had a significantly increased GlcNAc substitution of the WTA, but to a lesser degree than S. aureus VC40, and S. aureus VC40R was on par with the VSSA strain S. aureus NCTC8325. Regarding the d-alanine content, neither S. aureus VC40R nor S. aureus VraS(VC40) had an increased substitution, and S. aureus VC40R even showed a decrease in d-alanine per mole phosphate. This result was because the revertant strain S. aureus VC40R had the same amount of d-alanine as the parental strain but a significantly higher phosphate content. Decoration with d-Ala esters confers a positive charge to the otherwise negatively charged WTA polymers (22). An increase in d-alanine content in the cell wall should therefore decrease the overall negative charge, which was tested with the cytochrome *c* binding assay. Indeed, S. aureus VC40 had a drastically decreased ability to bind cytochrome *c* (see Fig. S4 in the supplemental material).

### Phage typing indicates additional alterations of the WTA of S. aureus VC40.

Besides their involvement in PBP4 and AtlA activity, the WTAs are also epitopes for the human immune response and receptors for phage interaction (23, 24). In this regard, the glycosylation pattern of the WTA is crucial since *Podoviridae* phages require β-GlcNAc residues on the WTA for adsorption (25) and *Siphoviridae* phages adsorb to α-GlcNAc (24) and most probably also to β-GlcNAc residues (26). In contrast, *Myoviridae* attach to the anionic backbone of WTAs (24). Phage typing showed that all strains were susceptible to *Myoviridae* and that, therefore, the WTA of all strains was still accessible for phages. In addition, S. aureus RN4220Δ*mutS*, which has lost all integrated phages as well as restriction enzyme activity, was—unlike the other strains—susceptible to nearly all phages of the international phage set, which are all *Siphoviridae* (Table 2). In stark contrast, apart from *Myoviridae*, the VISA strain S. aureus VC40 was infected only by *Podoviridae* and S. aureus VC40R showed lysis only with some *Siphoviridae*. The other strain pair S. aureus NCTC8325 and S. aureus VraS(VC40) did not differ significantly with regard to their susceptibility to the different phage families. In conclusion, the results indicated that in contrast to the parental strain and the revertant strain S. aureus VC40R, S. aureus VC40 may possess β-glycosylated WTAs.

**TABLE 2:** Phage typing with the international phage set and selected *Myoviridae* and *Podoviridae* phages

Strain or phage	Lysis result^*a*^ by phage type																												
	*Siphoviridae*																							*Myoviridae*			*Podoviridae*		
	29	52	52A	79	80	3A	3C	55	71	6	42E	47	53	54	75	77	83A	84	85	94	96	81	95	K	SK311	812	P68	44	66
RN4220Δ*mutS*	+	+	+	+	+	+	+	+	+	+	+	+	+	+	+		+			+	+	+	+	+	+	+			
VC40																								+	+	+	+	+	+
VC40R		+										+		+	+					+				+	+	+			
NCTC8325	+									+		+		+	+	+	+	+	+				+	+	+	+	+	+	+
VraS(VC40)	+	+								+		+		+	+	+		+	+				+	+	+	+	+	+	+

a +, lysis.

### S. aureus VC40 has β-glycosylated wall teichoic acids.

**FIG 4:**
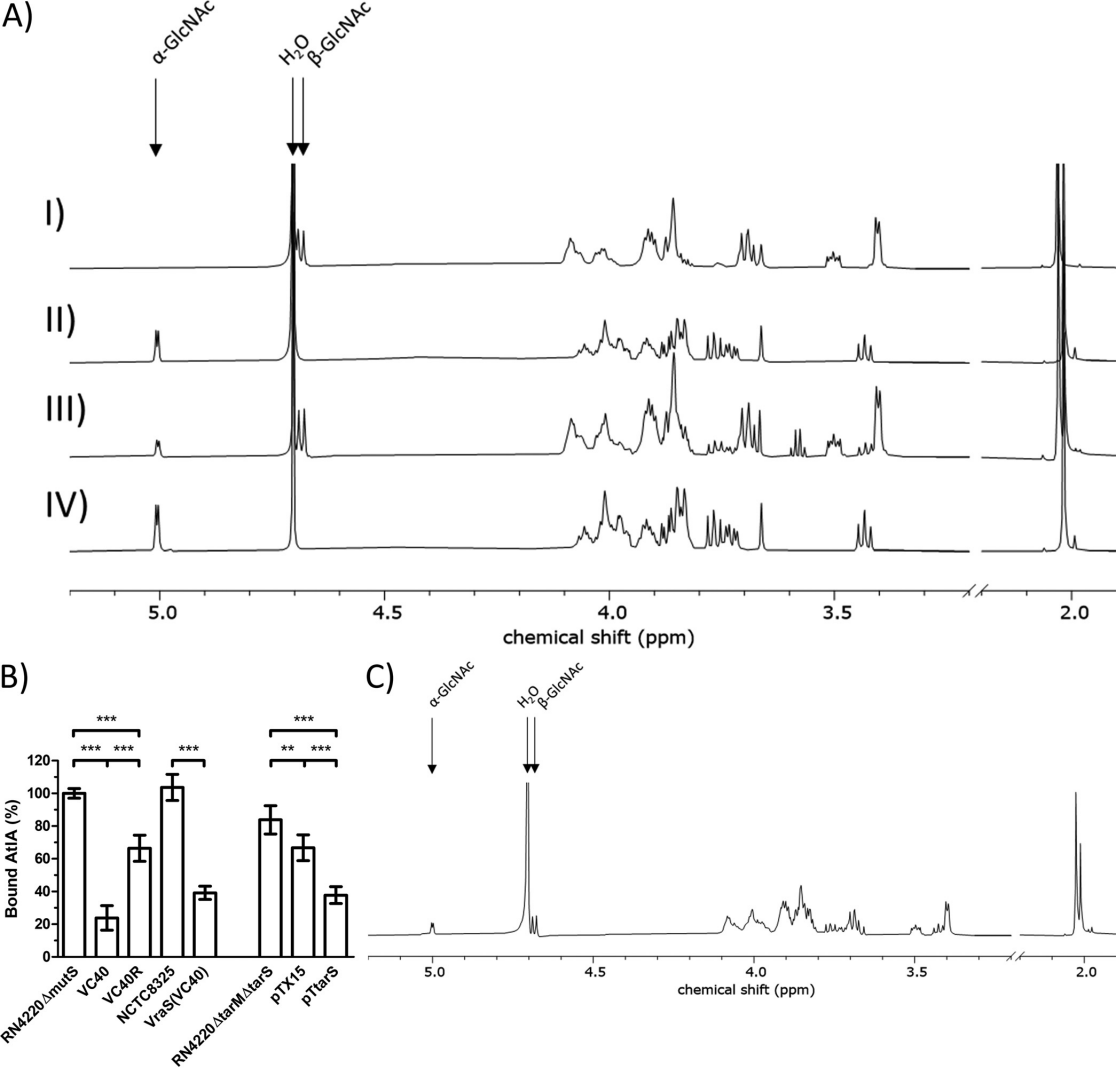
(A) ^1^H NMR spectra of purified WTA monomers from S. aureus RN4220Δ*tarM*Δ*tarS* pT*tarS* (I), S. aureus RN4220Δ*mutS* (II), S. aureus VC40 (III), and S. aureus VC40R (IV). Arrows indicate the ^1^H proton of the α-1,4-GlcNAc and β-1,4-GlcNAc residues on ribitol phosphate and the water peak, respectively. The figure shows a representative experiment; the analysis was performed in triplicate. (B) AtlA binding assay to determine the amount of bound AtlA on PGN with WTA after the removal of d-alanine substitutions. S. aureus RN4220Δ*mutS* set to 100%. (*, *P* < 0.05; **, *P* < 0.005; ***, *P* < 0.0005). (C) ^1^H NMR spectrum of purified WTA monomers from S. aureus VC40R grown in medium with 4% NaCl. Arrows indicate the ^1^H proton of the α-1,4-GlcNAc and β-1,4-GlcNAc residues on ribitol phosphate and the water peak, respectively. The figure shows a representative experiment, and the analysis was performed in triplicate.

To further investigate the glycosylation patterns, an NMR analysis of the WTAs of S. aureus RN4220Δ*mutS*, S. aureus VC40, and the revertant S. aureus VC40R was performed. A positive control for a strain harboring only β-glycosylated WTAs was represented by a double knockout of both glycosidase genes (*tarM* and *tarS*) harboring the plasmid pTX15 for the inducible expression of *tarS* in the presence of xylose, namely, S. aureus RN4220Δ*tarM*Δ*tarS* pT*tarS* (Fig. 4A). The spectra clearly showed the absence of β-1,4-GlcNAc residues for S. aureus RN4220Δ*mutS* and S. aureus VC40R. S. aureus VC40 had both types of glycosylated WTAs in a ratio of 1:3 in favor of β-1,4-GlcNAc. Whole-genome sequencing had indicated intact copies of *tarM* (α-1,4-GlcNAc) and *tarS* (β-1,4-GlcNAc) in S. aureus RN4220Δ*mutS* and S. aureus VC40 (27, 28), and the genome sequence analysis in this study also confirmed that S. aureus VC40R and S. aureus VraS(VC40) harbor intact copies of both enzymes. Interestingly, the S. aureus pT*tarS* strain did not show any difference in its vancomycin MIC in BHI and MH media compared with the empty vector control, irrespective of the presence of xylose.

### β-Glycosylated wall teichoic acids lead to a decreased binding affinity of AtlA to the cell wall.

The difference in the glycosylation pattern between S. aureus VC40 and its related strains could influence the susceptibility to autolysis. Therefore, an assay to determine the amount of AtlA bound to the cell walls was performed for all strains. A set amount of purified AtlA was incubated for 15 min with purified PGN of each strain, without d-Ala substitution on the WTA, as well as with buffer to serve as a positive control. Then, the suspensions were centrifuged and pasteurized M. luteus cells were added to the PGN-free supernatants. Lysis of cells by AtlA still present in the supernatant was observed and compared with the positive control (no cell walls) to estimate the content of unbound AtlA. S. aureus RN4220Δ*mutS* was used as a reference, and the amount of AtlA adsorbed from this supernatant was set to 100% (Fig. 4B) which amounted to 62% of the added AtlA. All three VISA cell wall fractions bound significantly less AtlA than their respective parent strains. The S. aureus VC40 cell wall had the lowest affinity to AtlA of all strains tested and adsorbed only about 23% compared with S. aureus RN4220Δ*mutS*. S. aureus VraS(VC40), which previously also had shown decreased autolysis and an altered autolysis profile (Fig. 2A and B), adsorbed the second smallest amount of bound AtlA. Although the revertant S. aureus VC40R had not exhibited decreased lysis (Fig. 2B), it bound only 66% of AtlA compared with S. aureus RN4220Δ*mutS*. Interestingly, the presence of only β-glycosylated WTA in the S. aureus pT*tarS* strain decreased the quantity of bound AtlA significantly compared with a strain deleted for both glycosylation enzymes TarS and TarM. A small but significant decrease was also seen for the empty vector control.

### Salt stress induces β-glycosylation of the wall teichoic acids and decreases the vancomycin susceptibility of the revertant strain S. aureus VC40R dramatically.

Recently, it was shown that the β-glycosylated WTA is favored at the expense of the α-glycosylated form when grown on medium with high salt concentrations (29). To see if salt stress and in turn an increased amount of β-1,4-GlcNAc substituted WTA influence vancomycin susceptibility, an MIC in medium with 4% NaCl was performed. Interestingly, most strains did not show any difference in either medium (Table 3). Most notably, the S. aureus VC40R strain quadrupled the MIC of vancomycin in both media upon the addition of salt. It reached the same vancomycin susceptibility in MH medium as its progenitor S. aureus VC40. In order to verify the incorporation of β-glycosylated sugars in the presence of salt, the WTA was purified from S. aureus VC40R that had been incubated in medium with 4% NaCl and analyzed by NMR. The spectrum showed both peaks for α-1,4-GlcNAc and β-1,4-GlcNAc (Fig. 4C). In addition, the strain also displayed susceptibility to *Podoviridae* when incubated on agar containing 4% NaCl (results not shown).

**TABLE 3:** Susceptibility testing for vancomycin of different strains after 48 h in BHI and MH medium with and without the addition of 4% NaCl^*a*^

Strain	Susceptibility result (μg/ml) by medium			
	BHI	BHI + 4% NaCl	MH	MH + 4 % NaCl
RN4220Δ*mutS*	2	2	1	1
VC40	64	64	16	16
VC40R	4	32	2	16
VraS(VC40)	4	4	4	4

a Shows a typical result of one of three experiments.

## DISCUSSION

The three main characteristics of most clinically relevant and laboratory-generated VISA strains are an increased cell wall diameter with a decreased cross-linking, a decreased autolysis, and attenuated virulence. These characteristics are well documented for a large number of isolates (11, 12), but the underlying mechanisms need further research. Indeed, the three VISA strains of this study exhibit these features to some extent as well.

The most important characteristic for the decreased vancomycin susceptibility is the cell wall of S. aureus. Vancomycin forms hydrogen bonds with the d-Ala-d-Ala terminus of lipid II and/or the free PGN pentapeptides (12). An increase in cell wall thickness and a decrease in cross-linking lead to an increase in free d-Ala-d-Ala residues in the PGN. Therefore, the ability to absorb vancomycin into the cell wall is directly proportional to these metrics. In turn, this binding of vancomycin prevents the diffusion of vancomycin to lipid II in the cell wall biosynthetic complex (15), which is the reason for the high vancomycin MIC of S. aureus VC40. The cost of a decrease in PGN cross-linking for S. aureus cells is a reduction in cell wall stability caused by a larger mesh size (30). To balance the low level of peptide bridges, the cells need to decrease their autolysis. Here, it is shown that S. aureus VC40 counters the decrease in cross-linking by a modification of wall teichoic acids, especially by β-glycosylation, to hinder AtlA activity and thus protect its fragile PGN.

A decreased autolysis, because of an altered autolysin profile, specific mutations, a decreased expression, or a decreased affinity toward the PGN, leads to improper cell separation and an increased cell wall thickness. Autolysins in S. aureus are regulated via the TCS WalRK and most likely processed through proteases regulated by the *msaABCR* operon (31, 32). WalK carries a mutation in S. aureus VC40 (6). Interestingly, real-time PCR measurements of *ssaA*, *lytM*, *atlA*, and *sceD* transcripts suggested that most autolysins in S. aureus VC40 including AtlA are upregulated (6). It seems that an altered processing of AtlA, potentially via Ssp serine and cysteine proteases, which are negatively regulated by *msaABCR* (32), are responsible for the autolysin profile in the zymogram. This factor, together with the reduced affinity of the autolysin toward the PGN due to the β-glycosylated WTA, as well as an increase in overall WTA concentration, reduced the autolysis in S. aureus VC40, despite the increase in autolysin expression.

The role of wall teichoic acids in vancomycin resistance is incompletely understood. In 2003, Sieradzki and Tomasz (33) found a quantitative or qualitative change in the WTA in the vancomycin intermediate progeny of the clinical isolate JH1. All VISA strains in our study had an increased WTA concentration with an increased GlcNAc substitution and, in the case of S. aureus VC40, also an increase in d-alanine. Increased wall teichoic acid production and d-alanylation are also common among daptomycin-resistant clinical isolates, which are often cross-resistant to vancomycin (34). This finding also applies to S. aureus VC40 which shows cross-resistance to daptomycin (6). In a paper by Peschel et al. (35) the strains lacking d-alanine substitutions on the WTA exhibited an increased susceptibility to glycopeptide antibiotics. d-Alanylation of the WTA is governed by the *dlt* operon, which is GraSR dependent. With an increase in positively charged d-alanine residues, the cell wall is less susceptible toward cationic antimicrobials since the d-alanine substitution impedes the ionic interaction of the cationic vancomycin with the negatively charged teichoic acids (36). This charge-dependent decrease in binding of vancomycin could also play a role in the S. aureus VC40 phenotype. Interestingly, this strain has an additional mutation in *mprF*, coding for a membrane protein that modifies anionic phosphatidylglycerol with l-lysine and thereby diminishing the affinity of the cytoplasmic membrane for cationic antimicrobial peptides. Through allelic exchange, it could be demonstrated that this mutation alone had no effect on the adsorption of cytochrome *c* to the cell envelope of S. aureus VC40, which was therefore influenced solely by the WTA d-alanylation (see Fig. S5 in the supplemental material).

The checkerboard MIC assay underlines the importance of the WTA for the reduced susceptibility toward vancomycin for S. aureus VC40. WTAs also have a connection to methicillin resistance in strains with PBP2a. Inhibiting the first enzyme of WTA synthesis TarO sensitizes MRSA to β-lactams (37). However, a synergistic effect of vancomycin and tunicamycin on any of the MRSA tested was not reported. Brown et al. (38) further showed that the glycosylation pattern is important for the sensitization to β-lactams. In S. aureus VC40 and its revertant strain S. aureus VC40R, the glycosylation of WTA also seemed to have a major impact on the phenotype. Both strains showed a strong reduction in phage susceptibility. A contrast in phage typeability in isogenic VISA/VSSA pairs was also observed by Gustafson et al. (39). In our study, the reason for the absence of phage typeability of S. aureus VC40 was the difference in the glycosylation pattern of the WTA. *Myoviridae* recognize the backbone of the WTA and show that this polymer is still accessible for phages in all strains. *Podoviridae* attach only to cell walls with β-glycosylated WTA (40), which is the case for S. aureus VC40, S. aureus NCTC8325, and S. aureus VraS(VC40). The NMR analysis of S. aureus VC40 and its relatives confirmed these results.

Glycosylation of S. aureus wall teichoic acids is influenced by the salt concentration in the environment. The β-GlcNAc anomer is integrated preferentially into the WTA under high salinity at the expense of the α-GlcNAc anomer (29). On the other hand, salt stress decreases the transcription of the *dlt* operon and in turn the d-alanylation of the WTA as well (41). Here, it could be shown that 4% NaCl leads to the incorporation of the β-1,4-GlcNAc anomer in the S. aureus VC40R TA. S. aureus NCTC8325 and S. aureus VraS(VC40) may also integrate a mixture of alpha and beta sugars in the absence of salt stress, as both are lysed by *Podoviridae* as well as *Siphoviridae*. The incorporation of β-1,4-GlcNAc into the WTA resulted in a stunning decrease of the vancomycin susceptibility in the revertant strain S. aureus VC40R. In contrast, growth of the parental strains S. aureus RN4220Δ*mutS* under high salt concentrations and the overexpression of TarS, which should both induce the β-glycosylation of WTA, did not alter the susceptibility toward vancomycin in these strains. Likewise, S. aureus VraS(VC40) displayed no decreased vancomycin susceptibility in medium with NaCl. However, this strain already contained β-glycosylated WTA, as shown from the lysis by *Podoviridae*. From this finding, it can be concluded that the β-glycosylation of the WTA leads only to a decrease in vancomycin susceptibility when other VISA characteristics, like the *walK* mutation, low cross-linking, and an increased cell wall diameter, are already present. The effect of osmolarity on the vancomycin MIC is noted in some papers (42). Goldstein et al. (43) found that 14 clinical VISA isolates increased their vancomycin MIC in BHI medium with 4% NaCl up to 3-fold compared with that of medium without salt. Other MRSA and MSSA control strains as well as the VISA strain Mu50 and hVISA strain Mu3 showed no effect. Interestingly, clinical VISA strains of the clonal complex 5, like Mu50, Mu3, JH9, and others, often possess only the *tarS* gene, which is responsible for the β-glycosylation of WTA (44). In contrast, Howden et al. (45) showed in microarray experiments with clinical strain pairs that had acquired resistance during treatment with vancomycin that three of five VISA strains had significantly downregulated *tarM*, which was responsible for α-glycosylation of the WTA, compared with their VSSA counterparts. It is established that wall teichoic acids are spatial and temporal regulators of PBP4 and AtlA activity (16, 17). Both enzymes operate at the cell division septum, which is devoid of mature WTA. Biswas et al. (46) suggested that the negatively charged WTAs retain protons in the cell wall, which creates an acidic environment that leads to low AtlA activity. Here, it was shown that the substitution of WTA with β-1,4-GlcNAc leads to a reduced binding of AtlA to the cell wall. This result presents a novel connection between autolysis, WTA glycosylation, and reduced vancomycin susceptibility in VISA strains and corresponds to early observations indicating that changes in the cell wall biochemistry of laboratory and clinical VISA might affect the activity of AtlA (47). However, this mechanism may be specific only for some VISA strains and depend on additional mutations present in strains S. aureus VC40 and S. aureus VC40R. Additionally, it must be noted that the resistance level of S. aureus VC40 is far higher than that of clinical isolates.

The strain S. aureus VC40 has two mutations in *vraS*, encoding a histidine kinase of a cell wall damage-sensing TCS. VraS is a hot spot for mutations in clinical VISA strains, although double mutations are rare and have not been described before. Only three clinical teicoplanin-resistant strains from Kato et al. (48) have double mutations in VraS (V138M/V236A, I5N/P246S, and A172T/F321L). These mutations in S. aureus VC40 lead to a steady elevated expression of VraSR-regulated genes like *vraS*, *lytM*, and *sgtB*. The introduction of these mutations into the S. aureus NCTC8325 background resulted in the same effect (6). In S. aureus VC40R, the additional mutation in *vraR* abrogates phosphorylation and in turn would decrease the expression of regulated genes. Therefore, any differences from the wild type, shared by S. aureus VC40 and S. aureus VraS(VC40) and reversed in the S. aureus VC40R strain, have the potential to be linked to the *vraSR* TCS. In this sense, the reduced autolysis and the increased substitution of the WTA with sugars could be associated with the altered *vraSR* activity.

In conclusion, compositional changes in the WTAs are observed in many clinical and laboratory VISA strains, either through quantitative modifications of WTA concentration or d-alanylation (33–35). Here, it is shown for the first time that the glycosylation pattern of wall teichoic acids may be another facet of decreasing vancomycin susceptibility. The switch to a mixture of α- and β-GlcNAc substitutions on the WTA led to an 8-fold increase in MIC in the revertant S. aureus VC40R and could be due to the decrease in AtlA binding to the cell wall and subsequent decreased autolysis.

## MATERIALS AND METHODS

### Bacterial strains, plasmids, and growth conditions.

All bacterial strains are listed in Table 4. S. aureus strains were grown in tryptic soy broth (TSB; (Oxoid, Wesel, Germany) or brain heart infusion broth (BHI; Oxoid) at 37°C, unless indicated otherwise.

**TABLE 4:** Bacterial strains and plasmids used in this study

Strain or plasmid	Description	Source or reference
Strains		
Staphylococcus aureus		
NCTC8325	Laboratory strain, MSSA, *agr*^+^	61
RN4220	Transformable laboratory strain, MSSA, *agr^−^*	27
RN4220Δ*mutS*	*mutS* knockout mutant of S. aureus RN4220, mutator phenotype, *agr^−^*	18
VC40	Vancomycin-resistant mutant of S. aureus RN4220Δ*mutS*, *agr*^+^	6
VC40R	Spontaneous revertant of S. aureus VC40 with additional exchange (M54T) in VraR, *agr*^+^	This study
VraS(VC40)	Derivative of strain S. aureus NCTC8325 harboring the D242G and L114S exchanges of S. aureus VC40 in VraS, *agr*^+^	6
RN4220Δ*tarM*Δ*tarS*	Deletion mutant for both WTA glycosylases of S. aureus RN4220, *agr^−^*	62
Escherichia coli		
JM109	E. coli cloning host	63
DC10B	Universal staphylococcal cloning host	64
M15	Strain for recombinant gene expression	65
BL21(DE3) pREP*groESL*	Strain for recombinant gene expression, λDE3 lysogen, T7 RNA polymerase gene, constitutive expression of chaperons	49
Micrococcus luteus		
ATCC 4698	Sensitive indicator organism	ATCC
Plasmids		
pTX15	Xylose-inducible staphylococcal expression vector, Tet^r^	66
pT*tarS*	pTX15 plasmid for xylose-inducible expression of *tarS*	This study
pQE-32_AtlA	Overexpression plasmid for AtlA without leader sequence	This study
pET22bΔ*pelB*_VraR	Overexpression plasmid for VraR	This study
pET22bΔ*pelB*_VraR(M54T)	Overexpression plasmid for VraR(M54T)	This study
pET22bΔ*pelB*_VraS	Overexpression plasmid for VraS	This study

### Molecular cloning procedures and protein purification.

The primers used for molecular cloning are listed in Table S1 in the supplemental material. All genes were amplified from S. aureus NCTC8325 genomic DNA. To generate a strain for the inducible expression of *tarS* under the control of the xylose-inducible *xylA* promoter and the *xylR* repressor, the gene was ligated into the pTX15 vector (49) using BamHI and *Nar*I. The recombinant plasmid was shuttled into S. aureus RN4220Δ*tarM*Δ*tarS* by electroporation. Tetracycline (25 μg/ml) was used as a selection marker. Transcription of the inducible gene was achieved by supplementation with 0.5% xylose (Merck, Darmstadt, Germany).

For the C-terminal His-Tag fusion proteins of the response regulator VraR and the cognate histidine kinase VraS, the genes were cloned into the pET22bΔ*pelB* vector (50). Each gene was ligated into the vector using NcoI and XhoI/SalI and afterward introduced into Escherichia coli JM109 through CaCl_2_-transformation and E. coli BL21(DE) through electroporation. Following the cloning of pET22bΔ*pelB*_vraR, the point mutation of the revertant S. aureus VC40R was introduced into the plasmid using the QuikChange Lightning site-directed mutagenesis kit from Agilent Technologies (Santa Clara, USA) according to the manufacturer’s instructions. To avoid the formation of inclusion bodies, the expression strain E. coli BL21(DE) also contained the pREP4*groESL* plasmid (49). Kanamycin (25 μg/ml) and ampicillin (40 μg/ml) were used as selection markers.

For the N-terminal His-Tag fusion protein of the major autolysin of S. aureus AtlA, the gene was cloned into the pQE-32 (Qiagen, Hilden, Germany) vector. To this end, *atlA* was ligated into the vector using BamHI and XhoI. The vector was then shuttled into E. coli DC10B and subsequently introduced into E. coli M15 (Qiagen) via CaCl_2_ transformation. Kanamycin (25 μg/ml) and ampicillin (40 μg/ml) were used as selection markers.

Expression, purification, and dialysis of proteins were performed according to Türck and Bierbaum (49) using 1 mM isopropyl-β-d-thiogalactopyranoside (IPTG; Thermo Scientific, Schwerte, Germany) to induce protein expression.

### Phosphorylation of VraR.

The phosphorylation of VraR and the VraR(M54T) variant with the cognate kinase VraS was performed essentially as described in Gajdiss et al. (51). Afterward, the phosphorylation was evaluated by 12.5% Phos-Tag (Wako Chemicals, Neuss, Germany) SDS-PAGE (52).

### Peptidoglycan purification and muropeptide analysis.

The peptidoglycan purification and UPLC analysis of a 6-ml overnight culture were performed as described previously (53). The cross-linking was calculated from the peak areas according to the following formula (54):

$$\frac{\left(1/2\times \text{dimers}\right)\;+\;\left(2/3\times \text{trimers}\right)\;+\;\left(3/4\times \text{tetramers}\right)\;+\;\left(9/10\times \text{multimers}\right)}{\text{sum of all areas}}$$

### Transmission electron microscopy.

The preparation of cells and transmission electron microscopy were carried out as detailed in Berscheid et al. (6) using an EM 900 transmission electron microscope (TEM; Carl Zeiss Microscopy, Oberkochen, Germany) at magnifications of 30,000- to 50,000-fold.

### Antimicrobial susceptibility testing and phage typing.

MIC testing was performed in Mueller-Hinton broth (Oxoid) or BHI broth using the colony suspension and broth microdilution method in 96-well microplates with an inoculum of 10^5^ CFU/ml as described by Wiegand et al. (55) and according to CLSI standards.

Phage typing was carried out with the international set of phages as well as three *Myoviridae* (ϕK, ϕSK311, and ϕ812) and three *Podoviridae* (ϕP68, ϕ66, and ϕ44) at routine test dilutions according to the subcommittee on phage typing of staphylococci of the International Association of Microbiological Societies.

### Preparation of cell walls, purification of WTA, and analytical methods.

For the isolation of cell walls, the strains were incubated in 50-ml BHI overnight and then harvested. The pellet was washed with 0.5 M citric acid buffer (pH 3), and the cells were lysed with glass beads in a Precellys 24 homogenizer (Peqlab, Erlangen, Germany) at 6,000 rpm. A low pH (pH 3) was used to stabilize the base-labile d-Ala residues on the teichoic acids. The lysate was centrifuged twice at low speed (3,000 × *g* for 5 min at room temperature [RT]) to sediment cell debris. Then, the supernatant was centrifuged at high speed (16,000 × *g* for 20 min at 4°C) to isolate the cell walls. These samples were then resuspended in 0.5 M citric acid buffer (pH 3) with 2% SDS (Serva, Heidelberg, Germany) and incubated at 37°C with aeration (50 rpm) for 2 h. The cell walls were then washed 4 times with 20 mM sodium acetate buffer (pH 4.75) and freeze-dried.

The WTA purification was based on Covas et al. (56). Briefly, 20 ml of an overnight culture in BHI was harvested, washed with 50 mM morpholineethanesulfonic acid (MES; Sigma-Aldrich) buffer (pH 6.5; buffer 1), and boiled for 1 hour in 50 mM MES buffer (pH 6.5) with 4% SDS (buffer 2). Next, the samples were washed successively with buffer 2, then with 50 mM MES (pH 6.5) with 2% NaCl (buffer 3), and finally with buffer 1 and incubated for 4 hours at 50°C with aeration (180 rpm) in 20 mM Tris (pH 8) with 0.5% SDS and 20 μg/ml proteinase K (Sigma-Aldrich). Then, the samples were washed with buffer 3 and washed thrice with H_2_O, before being resuspended in 0.1 M NaOH overnight at RT and aeration (180 rpm) to release the WTA. Finally, the supernatants were neutralized with 1 M HCl and freeze-dried.

d-Alanine content was estimated enzymatically by the method of Graßl (57) after liberation from isolated cell walls in 0.1 M NaOH at RT overnight. For the determination of d-glucosamine, the purified WTAs were boiled for 2 hours in 4 M HCl, neutralized with 2 M NaOH, and analyzed with a d-glucosamine assay kit (Megazyme, Wicklow, Ireland). The inorganic phosphate content was quantified for both isolations according to Rouser et al. (58).

### WTA preparation for NMR analysis.

WTA was extracted according to Kho and Meredith (59) with minor modifications. Briefly, S. aureus strains were grown overnight at 37°C in 500-ml TSB, harvested, washed once with 50 mM MES buffer (pH 6.5), and resuspended in 20-ml 50 mM MES buffer (pH 6.5) with 4% SDS. The samples were then boiled for 1 hour and centrifuged. Then, the pellets were washed once with phosphate-buffered saline (PBS), thrice with 0.5% SDS, and thrice with H_2_O and incubated in H_2_O at 60°C for 30 minutes. The cells were centrifuged and washed once with 30-ml H_2_O and incubated overnight in 10 ml of a 0.2-mg/ml trypsin (Carl Roth, Karlsruhe, Germany) solution in 15 mM Tris-HCl (pH 7) at 37°C with aeration (180 rpm). After the trypsin digestion, the samples were washed once with 1 M Tris-HCl (pH 7), once with 1 M Tris-HCl, 1 M NaCl (pH 7), thereafter again once with 1 M Tris-HCl (pH 7) and thrice with H_2_O. To release the WTA, the pellets were then resuspended in 0.1 M NaOH and incubated overnight at RT with aeration (180 rpm). The samples were centrifuged and the supernatants were neutralized with 0.1 volumes of 3 M sodium acetate. To precipitate the WTA, 3 volumes of 95% ethanol were added, and the samples were incubated overnight at −20°C. The WTA-containing solutions were then centrifuged, and the pellets were washed six times with 95% ethanol. After the WTA pellets were air-dried, they were freeze-dried successively once in H_2_O and twice in D_2_O (Sigma-Aldrich). WTAs in D_2_O were then analyzed in an 1D H-NMR at 298 K and with 256 scans on a Bruker Avance III HD 700-MHz instrument.

### Purification of crude autolysin extract and analysis of autolysin activity.

Purification of autolysins and zymographic analysis were performed essentially according to Gajdiss et al. (60). S. aureus strains were grown in 100-ml TSB until exponential growth (optical density at 600 nm [OD_600_] of 1). Afterward, all further steps were performed at 4°C. The cells were harvested and washed with double-distilled water (ddH_2_O), and the autolysins were released by incubation in 50 mM Tris-HCl (pH 7) buffer with 3 M LiCl (Merck) for 1 hour on ice. The autolysin extracts were adjusted to represent an equal number of cells, based on optical density, for all cultures. Afterward, cells were removed by centrifugation, and the supernatants with the crude autolysin extract were stored in 50% glycerol at −20°C.

The autolysin activity of the crude autolysin extracts were tested on peptidoglycans, which were isolated as described above. A PGN suspension was adjusted to an OD_600_ of 0.5 in 50 mM Tris-HCl and 150 mM NaCl (pH 7) and incubated at 37°C with approximately a 1/10 volume of autolysins prepared as described above. A control without autolysins was also used. The OD_600_ as an indicator of PGN digestion was determined every 15 minutes using a UVi Line 9400 photometer (Schott Instruments, Mainz, Germany). Additionally, autolysins were analyzed in a 10% SDS-acrylamide gel containing pasteurized Micrococcus luteus cells. The gel was washed extensively with H_2_O and incubated overnight at 37°C in zymogram buffer, consisting of 50 mM Tris-HCl, 10 mM CaCl_2_, 10 mM MgCl_2_, and 0.1% Triton X-100 (pH 7.5). To achieve a higher contrast, the gel was stained with 0.1% methylene blue (Merck) for 5 min.

Additionally, purified AtlA (2 μg) was tested against different PGN fractions, namely, pasteurized whole cells, PGN with WTA containing d-Ala substitutions, PGN with WTA without d-Ala substitutions, and PGN without WTA. Pasteurized S. aureus cells were prepared from an overnight culture, which was incubated at 80°C for 20 min in ddH_2_O. PGN with WTA and d-Ala substitutions was purified as mentioned above. The release of d-Ala was achieved by incubation of purified PGN in 0.1 M Tris-HCl (pH 8.5) at RT overnight. Finally, PGN without WTA was purified according to Kühner et al. (53). The purified AtlA was also used to test the binding capacity of the respective PGN for AtlA. To this end, the PGN with WTA without the d-Ala substitution was adjusted to an OD_600_ of 0.3 in 50 mM Tris-HCl and 150 mM NaCl (pH 7) and incubated with 0.2 μg/ml AtlA for 15 min at 37°C. Then, the PGN was sedimented (21,000 × *g* for 5 min at RT), and pasteurized M. luteus cells were adjusted to an OD_600_ of 0.5 in the supernatant. The extent of cell wall degradation of M. luteus was directly proportional to the unbound autolysin contained in the supernatant and could be quantified with a positive control consisting of 0.2-μg/ml AtlA. Autolysin activity was measured as described above.

### Genome sequence analysis and SNP identification.

Genomic DNA from overnight cultures of S. aureus strains VC40R and VraS(VC40) was purified using the MasterPure Gram-positive DNA purification kit (Epicentre Biotechnologies, Madison, USA). Shotgun libraries with an insert size of approximately 300 bp were generated by fragmentation and end repair of DNA (Eurofins Genomics GmbH, Cologne, Germany). The libraries were sequenced on Illumina MiSeq (v2 chemistry) instrument, and the obtained reads were mapped on the genomes of the respective parent stains S. aureus VC40 (NCBI GenBank accession number CP003033) and S. aureus NCTC8325 (NCBI GenBank accession number NC_007795). Detection of single nucleotide polymorphisms (SNPs), insertions, and deletions was performed using the VarScan (v2.3.5) software (Eurofins Genomics GmbH).

### Statistical analysis.

All experiments resulting in collectible data were performed at least in triplicate. The statistical significance between mean values was determined by an unpaired Student’s *t* test with a confidence interval of 95% using Prism (GraphPad Software, San Diego, USA).

### Data availability.

Illumina sequencing reads of S. aureus VC40R and S. aureus VraS(VC40) have been submitted at the ENA under study accession number PRJEB43609.

## References

[1] Becker K, Schaumburg F, Fegeler C, Friedrich AW, Köck R, Prevalence of Multiresistant Microorganisms PMM Study. 2017. *Staphylococcus aureus* from the German general population is highly diverse. Int J Med Microbiol 307:21–27. doi:10.1016/j.ijmm.2016.11.007. 28017539

[2] Levine DP. 2006. Vancomycin: a history. Clin Infect Dis 42:S5–S12. doi:10.1086/491709. 16323120

[3] Hiramatsu K, Hanaki H, Ino T, Yabuta K, Oguri T, Tenover FC. 1997. Methicillin-resistant *Staphylococcus aureus* clinical strain with reduced vancomycin susceptibility. J Antimicrob Chemother 40:135–136. doi:10.1093/jac/40.1.135. 9249217

[4] Howden BP, Peleg AY, Stinear TP. 2014. The evolution of vancomycin intermediate *Staphylococcus aureus* (VISA) and heterogenous-VISA. Infect Genet Evol 21:575–582. doi:10.1016/j.meegid.2013.03.047. 23567819

[5] Howden BP, McEvoy CRE, Allen DL, Chua K, Gao W, Harrison PF, Bell J, Coombs G, Bennett-Wood V, Porter JL, Robins-Browne R, Davies JK, Seemann T, Stinear TP. 2011. Evolution of multidrug resistance during *Staphylococcus aureus* infection involves mutation of the essential two component regulator WalKR. PLoS Pathog 7:e1002359. doi:10.1371/journal.ppat.1002359. 22102812 PMC3213104

[6] Berscheid A, François P, Strittmatter A, Gottschalk G, Schrenzel J, Sass P, Bierbaum G. 2014. Generation of a vancomycin-intermediate *Staphylococcus aureus* (VISA) strain by two amino acid exchanges in VraS. J Antimicrob Chemother 69:3190–3198. doi:10.1093/jac/dku297. 25103491

[7] Neoh HM, Cui L, Yuzawa H, Takeuchi F, Matsuo M, Hiramatsu K. 2008. Mutated response regulator *graR* is responsible for phenotypic conversion of *Staphylococcus aureus* from heterogeneous vancomycin-intermediate resistance to vancomycin-intermediate resistance. Antimicrob Agents Chemother 52:45–53. doi:10.1128/AAC.00534-07. 17954695 PMC2223914

[8] Cui L, Isii T, Fukuda M, Ochiai T, Neoh HM, Camargo I, Watanabe Y, Shoji M, Hishinuma T, Hiramatsu K. 2010. An RpoB mutation confers dual heteroresistance to daptomycin and vancomycin in *Staphylococcus aureus*. Antimicrob Agents Chemother 54:5222–5233. doi:10.1128/AAC.00437-10. 20837752 PMC2981288

[9] Katayama Y, Sekine M, Hishinuma T, Aiba Y, Hiramatsu K. 2016. Complete reconstitution of the vancomycin-intermediate *Staphylococcus aureus* phenotype of strain Mu50 in vancomycin-susceptible *S. aureus*. Antimicrob Agents Chemother 60:3730–3742. doi:10.1128/AAC.00420-16. 27067329 PMC4879404

[10] Shoji M, Cui L, Iizuka R, Komoto A, Neoh HM, Watanabe Y, Hishinuma T, Hiramatsu K. 2011. *walK* and *clpP* mutations confer reduced vancomycin susceptibility in *Staphylococcus aureus*. Antimicrob Agents Chemother 55:3870–3881. doi:10.1128/AAC.01563-10. 21628539 PMC3147622

[11] Hu Q, Peng H, Rao X. 2016. Molecular events for promotion of vancomycin resistance in vancomycin intermediate *Staphylococcus aureus*. Front Microbiol 7:1601. doi:10.3389/fmicb.2016.01601. 27790199 PMC5062060

[12] McGuinness WA, Malachowa N, DeLeo FR. 2017. Vancomycin resistance in Staphylococcus aureus. Yale J Biol Med 90:269–281. 28656013 PMC5482303

[13] Cui L, Murakami H, Kuwahara-Arai K, Hanaki H, Hiramatsu K. 2000. Contribution of a thickened cell wall and its glutamine nonamidated component to the vancomycin resistance expressed by *Staphylococcus aureus* Mu50. Antimicrob Agents Chemother 44:2276–2285. doi:10.1128/AAC.44.9.2276-2285.2000. 10952568 PMC90058

[14] Reipert A, Ehlert K, Kast T, Bierbaum G. 2003. Morphological and genetic differences in two isogenic *Staphylococcus aureus* strains with decreased susceptibilities to vancomycin. Antimicrob Agents Chemother 47:568–576. doi:10.1128/AAC.47.2.568-576.2003. 12543661 PMC151770

[15] Cui L, Iwamoto A, Lian JQ, Neoh HM, Maruyama T, Horikawa Y, Hiramatsu K. 2006. Novel mechanism of antibiotic resistance originating in vancomycin-intermediate *Staphylococcus aureus*. Antimicrob Agents Chemother 50:428–438. doi:10.1128/AAC.50.2.428-438.2006. 16436693 PMC1366884

[16] Atilano ML, Pereira PM, Yates J, Reed P, Veiga H, Pinho MG, Filipe SR. 2010. Teichoic acids are temporal and spatial regulators of peptidoglycan cross-linking in *Staphylococcus aureus*. Proc Natl Acad Sci USA 107:18991–18996. doi:10.1073/pnas.1004304107. 20944066 PMC2973906

[17] Schlag M, Biswas R, Krismer B, Kohler T, Zoll S, Yu W, Schwarz H, Peschel A, Götz F. 2010. Role of staphylococcal wall teichoic acid in targeting the major autolysin Atl. Mol Microbiol 75:864–873. doi:10.1111/j.1365-2958.2009.07007.x. 20105277

[18] Schaaff F, Reipert A, Bierbaum G. 2002. An elevated mutation frequency favors development of vancomycin resistance in *Staphylococcus aureus*. Antimicrob Agents Chemother 46:3540–3548. doi:10.1128/AAC.46.11.3540-3548.2002. 12384362 PMC128741

[19] Bem AE, Velikova N, Pellicer MT, van Baarlen P, Marina A, Wells JM. 2015. Bacterial histidine kinases as novel antibacterial drug targets. ACS Chem Biol 10:213–224. doi:10.1021/cb5007135. 25436989

[20] Tajbakhsh G, Golemi-Kotra D. 2019. The dimerization interface in VraR is essential for induction of the cell wall stress response in *Staphylococcus aureus*: a potential druggable target. BMC Microbiol 19:153. doi:10.1186/s12866-019-1529-0. 31277575 PMC6612188

[21] Bertsche U, Weidenmaier C, Kuehner D, Yang SJ, Baur S, Wanner S, Francois P, Schrenzel J, Yeaman MR, Bayer AS. 2011. Correlation of daptomycin resistance in a clinical *Staphylococcus aureus* strain with increased cell wall teichoic acid production and d-alanylation. Antimicrob Agents Chemother 55:3922–3928. doi:10.1128/AAC.01226-10. 21606222 PMC3147621

[22] Reichmann NT, Cassona CP, Gründling A. 2013. Revised mechanism of d-alanine incorporation into cell wall polymers in Gram-positive bacteria. Microbiology (Reading) 159:1868–1877. doi:10.1099/mic.0.069898-0. 23858088 PMC3783018

[23] Kurokawa K, Takahashi K, Lee BL. 2016. The staphylococcal surface-glycopolymer wall teichoic acid (WTA) is crucial for complement activation and immunological defense against *Staphylococcus aureus* infection. Immunobiology 221:1091–1101. doi:10.1016/j.imbio.2016.06.003. 27424796

[24] Xia G, Corrigan RM, Winstel V, Goerke C, Gründling A, Peschel A. 2011. Wall teichoic acid-dependent adsorption of staphylococcal siphovirus and myovirus. J Bacteriol 193:4006–4009. doi:10.1128/JB.01412-10. 21642458 PMC3147540

[25] Li X, Gerlach D, Du X, Larsen J, Stegger M, Kühner P, Peschel A, Xia G, Winstel V. 2015. An accessory wall teichoic acid glycosyltransferase protects *Staphylococcus aureus* from the lytic activity of Podoviridae. Sci Rep 5:17219. doi:10.1038/srep17219. 26596631 PMC4667565

[26] Li X, Koç C, Kühner P, Stierhof YD, Krismer B, Enright MC, Penadés JR, Wolz C, Stehle T, Cambillau C, Peschel A, Xia G. 2016. An essential role for the baseplate protein Gp45 in phage adsorption to *Staphylococcus aureus*. Sci Rep 6:26455. doi:10.1038/srep26455. 27212064 PMC4876445

[27] Berscheid A, Sass P, Weber-Lassalle K, Cheung AL, Bierbaum G. 2012. Revisiting the genomes of the *Staphylococcus aureus* strains NCTC 8325 and RN4220. Int J Med Microbiol 302:84–87. doi:10.1016/j.ijmm.2012.01.002. 22417616

[28] Sass P, Berscheid A, Jansen A, Oedenkoven M, Szekat C, Strittmatter A, Gottschalk G, Bierbaum G. 2012. Genome sequence of *Staphylococcus aureus* VC40, a vancomycin- and daptomycin-resistant strain, to study the genetics of development of resistance to currently applied last-resort antibiotics. J Bacteriol 194:2107–2108. doi:10.1128/JB.06631-11. 22461548 PMC3318483

[29] Mistretta N, Brossaud M, Telles F, Sanchez V, Talaga P, Rokbi B. 2019. Glycosylation of *Staphylococcus aureus* cell wall teichoic acid is influenced by environmental conditions. Sci Rep 9:3212. doi:10.1038/s41598-019-39929-1. 30824758 PMC6397182

[30] Loskill P, Pereira PM, Jung P, Bischoff M, Herrmann M, Pinho MG, Jacobs K. 2014. Reduction of the peptidoglycan crosslinking causes a decrease in stiffness of the *Staphylococcus aureus* cell envelope. Biophys J 107:1082–1089. doi:10.1016/j.bpj.2014.07.029. 25185544 PMC4156677

[31] Delauné A, Dubrac S, Blanchet C, Poupel O, Mäder U, Hiron A, Leduc A, Fitting C, Nicolas P, Cavaillon JM, Adib-Conquy M, Msadek T. 2012. The WalKR system controls major staphylococcal virulence genes and is involved in triggering the host inflammatory response. Infect Immun 80:3438–3453. doi:10.1128/IAI.00195-12. 22825451 PMC3457574

[32] Bibek GC, Sahukhal GS, Elasri MO. 2019. Role of the *msaABCR* operon in cell wall biosynthesis, autolysis, integrity, and antibiotic resistance in *Staphylococcus aureus*. Antimicrob Agents Chemother 63:e00680-19. doi:10.1128/AAC.00680-19. 31307991 PMC6761503

[33] Sieradzki K, Tomasz A. 2003. Alterations of cell wall structure and metabolism accompany reduced susceptibility to vancomycin in an isogenic series of clinical isolates of *Staphylococcus aureus*. J Bacteriol 185:7103–7110. doi:10.1128/JB.185.24.7103-7110.2003. 14645269 PMC296238

[34] Bertsche U, Yang SJ, Kuehner D, Wanner S, Mishra NN, Roth T, Nega M, Schneider A, Mayer C, Grau T, Bayer AS, Weidenmaier C. 2013. Increased cell wall teichoic acid production and D-alanylation are common phenotypes among daptomycin-resistant methicillin-resistant *Staphylococcus aureus* (MRSA) clinical isolates. PLoS One 8:e67398. doi:10.1371/journal.pone.0067398. 23785522 PMC3681945

[35] Peschel A, Vuong C, Otto M, Götz F. 2000. The d-alanine residues of *Staphylococcus aureus* teichoic acids alter the susceptibility to vancomycin and the activity of autolytic enzymes. Antimicrob Agents Chemother 44:2845–2847. doi:10.1128/AAC.44.10.2845-2847.2000. 10991869 PMC90160

[36] Peschel A, Otto M, Jack RW, Kalbacher H, Jung G, Götz F. 1999. Inactivation of the *dlt* operon in *Staphylococcus aureus* confers sensitivity to defensins, protegrins, and other antimicrobial peptides. J Biol Chem 274:8405–8410. doi:10.1074/jbc.274.13.8405. 10085071

[37] Campbell J, Singh AK, Santa Maria JP, Kim Y, Brown S, Swoboda JG, Mylonakis E, Wilkinson BJ, Walker S. 2011. Synthetic lethal compound combinations reveal a fundamental connection between wall teichoic acid and peptidoglycan biosyntheses in *Staphylococcus aureus*. ACS Chem Biol 6:106–116. doi:10.1021/cb100269f. 20961110 PMC3025082

[38] Brown S, Xia G, Luhachack LG, Campbell J, Meredith TC, Chen C, Winstel V, Gekeler C, Irazoqui JE, Peschel A, Walker S. 2012. Methicillin resistance in *Staphylococcus aureus* requires glycosylated wall teichoic acids. Proc Natl Acad Sci USA 109:18909–18914. doi:10.1073/pnas.1209126109. 23027967 PMC3503181

[39] Gustafson JE, O'Brien FG, Coombs GW, Malkowski MJ, Grubb WB, Pfeltz RF, Wilkinson BJ. 2003. Alterations in phage-typing patterns in vancomycin-intermediate *Staphylococcus aureus*. J Med Microbiol 52:711–714. doi:10.1099/jmm.0.05210-0. 12867567

[40] Ingmer H, Gerlach D, Wolz C. 2019. Temperate phages of *Staphylococcus aureus*. Microbiol Spectr 7:7.5.1. doi:10.1128/microbiolspec.GPP3-0058-2018. PMC10921950 31562736

[41] Koprivnjak T, Mlakar V, Swanson L, Fournier B, Peschel A, Weiss JP. 2006. Cation-induced transcriptional regulation of the *dlt* operon of *Staphylococcus aureus*. J Bacteriol 188:3622–3630. doi:10.1128/JB.188.10.3622-3630.2006. 16672616 PMC1482844

[42] Jung SI, Kiem S, Lee NY, Kim YS, Oh WS, Cho HL, Peck KR, Song JH. 2002. One-point population analysis and effect of osmolarity on detection of hetero-vancomycin-resistant *Staphylococcus aureus*. J Clin Microbiol 40:1493–1495. doi:10.1128/JCM.40.4.1493-1495.2002. 11923379 PMC140347

[43] Goldstein FW, Atoui R, Ben Ali A, Nguyen JC, Ly A, Kitzis MD. 2004. False synergy between vancomycin and β-lactams against glycopeptide-intermediate *Staphylococcus aureus* (GISA) caused by inappropriate testing methods. Clin Microbiol Infect 10:342–345. doi:10.1111/j.1198-743X.2004.00856.x. 15059127

[44] Winstel V, Xia G, Peschel A. 2014. Pathways and roles of wall teichoic acid glycosylation in *Staphylococcus aureus*. Int J Med Microbiol 304:215–221. doi:10.1016/j.ijmm.2013.10.009. 24365646

[45] Howden BP, Smith DJ, Mansell A, Johnson PDR, Ward PB, Stinear TP, Davies JK. 2008. Different bacterial gene expression patterns and attenuated host immune responses are associated with the evolution of low-level vancomycin resistance during persistent methicillin-resistant *Staphylococcus aureus* bacteriaemia. BMC Microbiol 8:39. doi:10.1186/1471-2180-8-39. 18304359 PMC2289824

[46] Biswas R, Martinez RE, Göhring N, Schlag M, Josten M, Xia G, Hegler F, Gekeler C, Gleske AK, Götz F, Sahl HG, Kappler A, Peschel A. 2012. Proton-binding capacity of *Staphylococcus aureus* wall teichoic acid and its role in controlling autolysin activity. PLoS One 7:e41415. doi:10.1371/journal.pone.0041415. 22911791 PMC3402425

[47] Boyle-Vavra S, Challapalli M, Daum RS. 2003. Resistance to autolysis in vancomycin-selected *Staphylococcus aureus* isolates precedes vancomycin-intermediate resistance. Antimicrob Agents Chemother 47:2036–2039. doi:10.1128/AAC.47.6.2036-2039.2003. 12760894 PMC155830

[48] Kato Y, Suzuki T, Ida T, Maebashi K. 2010. Genetic changes associated with glycopeptide resistance *in Staphylococcus aureus*: predominance of amino acid substitutions in YvqF/VraSR. J Antimicrob Chemother 65:37–45. doi:10.1093/jac/dkp394. 19889788 PMC2800785

[49] Türck M, Bierbaum G. 2012. Purification and activity testing of the full-length YycFGHI proteins of *Staphylococcus aureus*. PLoS One 7:e30403. doi:10.1371/journal.pone.0030403. 22276191 PMC3262814

[50] Sass P, Bierbaum G. 2007. Lytic activity of recombinant bacteriophage phi11 and phi12 endolysins on whole cells and biofilms of *Staphylococcus aureus*. Appl Environ Microbiol 73:347–352. doi:10.1128/AEM.01616-06. 17085695 PMC1797112

[51] Gajdiss M, Türck M, Bierbaum G. 2017. Bacterial histidine kinases: overexpression, purification, and inhibitor screen. Methods Mol Biol 1520:247–259. doi:10.1007/978-1-4939-6634-9_15. 27873257

[52] Kinoshita E, Kinoshita-Kikuta E, Takiyama K, Koike T. 2006. Phosphate-binding tag, a new tool to visualize phosphorylated proteins. Mol Cell Proteomics 5:749–757. doi:10.1074/mcp.T500024-MCP200. 16340016

[53] Kühner D, Stahl M, Demircioglu DD, Bertsche U. 2014. From cells to muropeptide structures in 24 h: peptidoglycan mapping by UPLC-MS. Sci Rep 4:7494. doi:10.1038/srep07494. 25510564 PMC4267204

[54] Glauner B, Höltje JV. 1990. Growth pattern of the murein sacculus of *Escherichia coli*. J Biol Chem 265:18988–18996. doi:10.1016/S0021-9258(17)30613-0. 2229056

[55] Wiegand I, Hilpert K, Hancock REW. 2008. Agar and broth dilution methods to determine the minimal inhibitory concentration (MIC) of antimicrobial substances. Nat Protoc 3:163–175. doi:10.1038/nprot.2007.521. 18274517

[56] Covas G, Vaz F, Henriques G, Pinho MG, Filipe SR. 2016. Analysis of cell wall teichoic acids in *Staphylococcus aureus*. Methods Mol Biol 1440:201–213. doi:10.1007/978-1-4939-3676-2_15. 27311674

[57] Graßl M. 1970. d-Alanin, p 1641–1644. *In* Bergmeyer HU. Methoden der enzymatischen analyse, 2nd ed. Verlag Chemie.

[58] Rouser G, Fkeischer S, Yamamoto A. 1970. Two dimensional then layer chromatographic separation of polar lipids and determination of phospholipids by phosphorus analysis of spots. Lipids 5:494–496. doi:10.1007/BF02531316. 5483450

[59] Kho K, Meredith T. 2018. Extraction and analysis of bacterial teichoic acids. Bio-protocol 8:e3078. doi:10.21769/BioProtoc.3078. 34532535 PMC8342068

[60] Gajdiss M, Monk IR, Bertsche U, Kienemund J, Funk T, Dietrich A, Hort M, Sib E, Stinear TP, Bierbaum G. 2020. YycH and YycI regulate expression of *Staphylococcus aureus* autolysins by activation of WalRK phosphorylation. Microorganisms 8:870. doi:10.3390/microorganisms8060870. 32526915 PMC7355866

[61] Novick RP, Richmond MH. 1965. Nature and interactions of the genetic elements governing penicillinase synthesis in *Staphylococcus aureus*. J Bacteriol 90:467–480. doi:10.1128/jb.90.2.467-480.1965. 14329463 PMC315668

[62] Winstel V, Liang C, Sanchez-Carballo P, Steglich M, Munar M, Bröker BM, Penadés JR, Nübel U, Holst O, Dandekar T, Peschel A, Xia G. 2013. Wall teichoic acid structure governs horizontal gene transfer between major bacterial pathogens. Nat Commun 4:2345. doi:10.1038/ncomms3345. 23965785 PMC3903184

[63] Yanisch-Perron C, Vieira J, Messing J. 1985. Improved M13 phage cloning vectors and host strains: nucleotide sequences of the M13mpl8 and pUC19 vectors. Gene 33:103–119. doi:10.1016/0378-1119(85)90120-9. 2985470

[64] Monk IR, Shah IM, Xu M, Tan MW, Foster TJ. 2012. Transforming the untransformable: application of direct transformation to manipulate genetically *Staphylococcus aureus* and *Staphylococcus epidermidis*. mBio 3:e00277-11. doi:10.1128/mBio.00277-11. 22434850 PMC3312211

[65] Villarejo MR, Zabin I. 1974. β-Galactosidase from termination and deletion mutant strains. J Bacteriol 120:466–474. doi:10.1128/jb.120.1.466-474.1974. 4607501 PMC245784

[66] Peschel A, Ottenwälder B, Götz F. 1996. Inducible production and cellular location of the epidermin biosynthetic enzyme EpiB using an improved staphylococcal expression system. FEMS Microbiol Lett 137:279–284. doi:10.1111/j.1574-6968.1996.tb08119.x. 8998998

