# Supplementary material for: The Role of β-Glycosylated Wall Teichoic Acids in the Reduction of Vancomycin Susceptibility in Vancomycin-Intermediate Staphylococcus aureus
Source: Microbiol Spectr. 2021 Oct 20;9(2):e00528-21. doi: 10.1128/Spectrum.00528-21 (PMC8528128; doi:10.1128/Spectrum.00528-21)
Supplement: SUPPLEMENTAL FILE 1 — Supplemental material. Download SPECTRUM00528-21_Supp_1_seq2.pdf, PDF file, 0.8 MB [file spectrum00528-21_supp_1_seq2.pdf]

Supporting Information

***S. aureus* cell membrane isolation and PBP detection**

For the membrane isolation, 2 L of a bacterial culture at OD_{600} 1 were harvested and washed with PBS buffer, pH 7.4. The pellet was lysed with glass beads in a homogenizer at maximum speed for 6 times 30 seconds, with 30 second intervals on ice in between. Cell debris was removed by centrifugation (4,000 g, 5 min, 4 °C). Afterwards the supernatant was centrifuged (225,000 g, 1 h, 4 °C) in a Sorvall Discovery M120 SE (Thermo Scientific) ultracentrifuge. The pellet was then resuspended in PBS and incubated with 5 µg/mL bocillin-FL (Invitrogen; Schwerte, Germany) for 30 min in the dark at RT. A portion of the membrane proteins (50 µL) was mixed with 4x Laemmli buffer and denatured at 90 °C for 5 min. The sample was separated in a 7.5 % SDS-PAGE and analyzed under UV-light without staining.

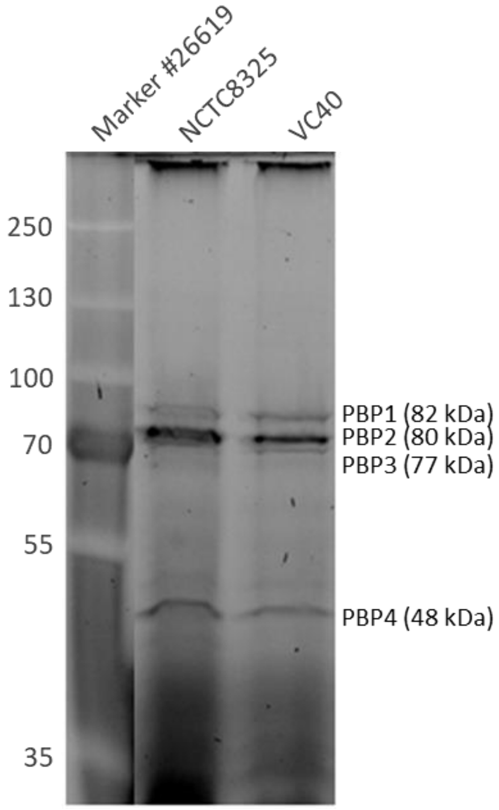

Figure S1 Detection of all four *S. aureus* PBPs in *S. aureus* NCTC8325 (positive control) and *S. aureus* VC40 with bocillin-FL in a 7.5 % SDS-PAGE.

Vancomycin binding assay

The vancomycin binding assay was used to determine the amount of vancomycin that bound to the cell wall of different *S. aureus* strains within a given time. Bacterial strains were incubated in 50 mL TSB until they reached an OD_{600} of 0.5. Then 30 µg/mL vancomycin was added, and samples were taken at time points 0, 2 and 4 hours. Samples were harvested at 10,000 g, 5 min, RT. The supernatants were pasteurized and aliquots of 50 µL (in triplicate) were loaded into prepared wells on a 0.6 % LB-agar plate (Oxoid), inoculated with 1 mL/L *Micrococcus luteus* culture (OD_{600} 0.3). Vancomycin standard solutions with concentrations between 1 – 30 µg/mL were used as a reference. The inhibition zones were measured after 24 h of incubation at 37 °C and the vancomycin concentrations in the supernatant were determined by comparison to reference wells.

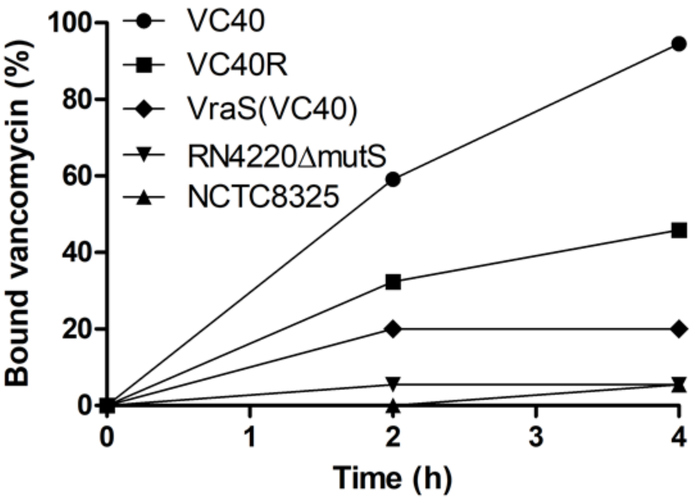

Figure S2 Vancomycin binding in a bioassay with different *S. aureus* strains over 4 hours of incubation with 30 µg/mL vancomycin. The figure shows a representative experiment, the assay was performed three times. The vancomycin concentration in the supernatant was estimated using *M. luteus*.

Checkerboard MIC

The synergism of vancomycin (Lilly Pharma; Giessen, Germany) and tunicamycin (Sigma-Aldrich), an antibiotic inhibiting the first stage in WTA synthesis, was evaluated by checkerboard MIC described elsewhere (1). The first antibiotic concentration increased from left to right and the second antibiotic concentration increased from top to bottom of the microplate. The fractional inhibitory concentration (FIC) was calculated based on the formulae $FIC_{Index}=FIC_A+FIC_B$, $FIC_A=MIC_{AB}/MIC_A$ and $FIC_B=MIC_{BA}/MIC_B$.

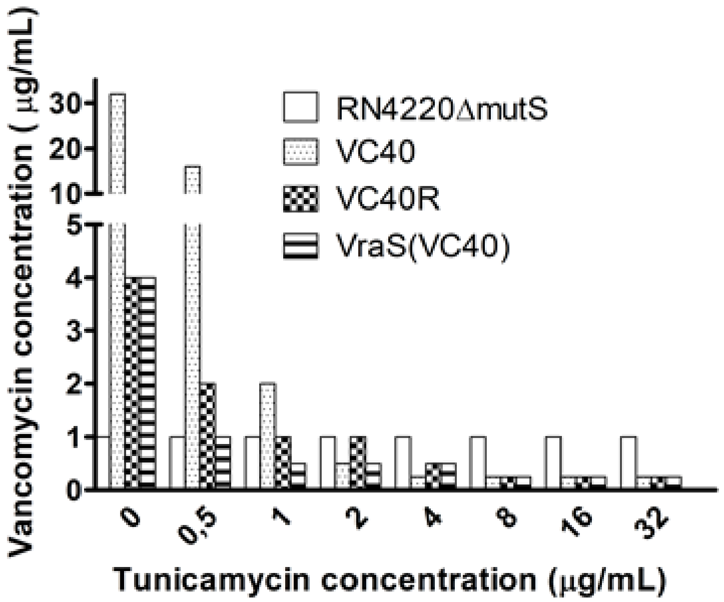

Figure S3 Checkerboard MIC of vancomycin and tunicamycin against *S. aureus* RN4220Δ*mutS*, *S. aureus* VC40, *S. aureus* VC40R and *S. aureus* VraS(VC40) in MH medium. The MIC was performed in triplicate and a representative assay is shown.

Analysis of cytochrome C binding

Changes in the charge of the cell wall, indicated by the amount of bound cytochrome C, were determined based on the method of Peschel et al. 1999 (2). In short, bacteria were grown to exponential phase (OD_{600} 1) and harvested by centrifugation. The pellet was washed twice with 20 mM MOPS (Sigma-Aldrich) buffer and adjusted to an OD_{600} of 7. Then 0.9 mL of culture was mixed with 0.1 mL 5 mg/mL cytochrome C (Sigma-Aldrich) and incubated for 10 minutes at RT. Samples were centrifuged and the OD_{530} of the supernatants was measured to determine the amount of cytochrome C bound to the cell walls.

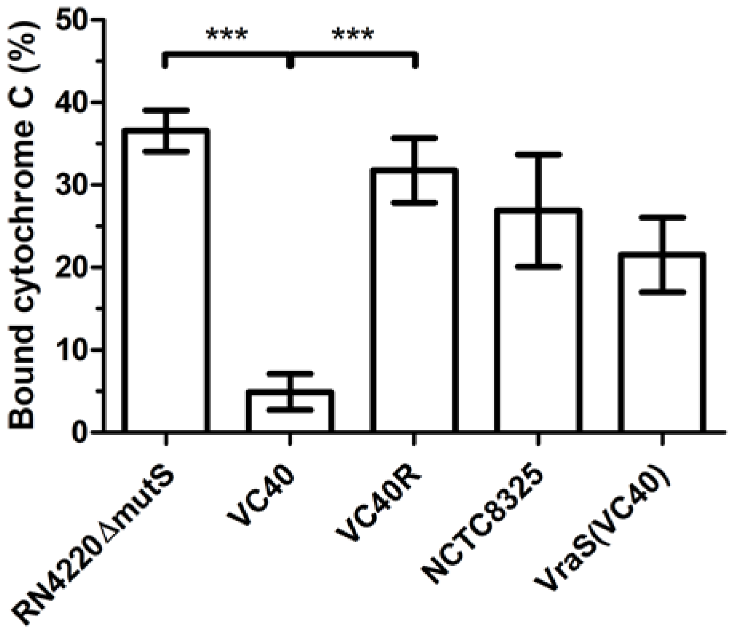

Figure S4 Cytochrome C binding assay showing percentage of bound compound to the cell wall of whole cells (*** p<0.0005).

S. aureus **NCTC8325 MprF(VC40)**

The reconstruction of the mutated *mprF* from *S. aureus* VC40 was performed according to Berscheid et al. 2014 (3), using the susceptible parent strain *S. aureus* NCTC8325 and the temperature-sensitive shuttle vector pMAD (4), that allows for markerless double homologous recombination. Afterwards the allelic exchange was confirmed by Sanger sequencing.

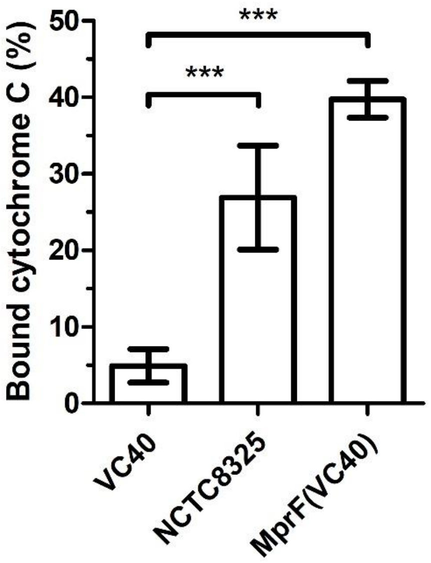

Figure S5 Cytochrome C binding assay showing percentage of bound compound to the cell envelope (*** p<0.0005).

Table S1 Oligonucleotide primers used in this study.

Primer	Sequence	Restriction Site
tarS_for	ATTAGGATCCGTGGGAGAGGTATAATGATG	*Bam*HI
tarS_rev	AGACGTGGCGCCCCTCAAATCATGTTGGCTCA	*Nar*I
vraR_for	GGACCATGGGTATGACGATTAAAGTATTGT	*Nco*I
vraR_rev	TACCTCGAGTTGAATTAAATTATGTTGGAA	*Xho*I
vraS_for	GTGCCATGGCGATGAACCACTACAT	*Nco*I
vraS_rev	AATGTCGACATCGTCATACGAATCC	*Sal*I
vraR(M54T)_for	CATGAGTTGAAGCCAGATTTAATTTTAACGGATTTACTTATGG ATGACA	
vraR(M54T)_rev	TGTCATCCATAAGTAAATCCGTTAAAATTAAATCTGGCTTCAA CTCATG	
AtlA_for	AAAAAGGATCCTTGCTGAGACGACACAAGATC	*Bam*HI
AtlA_rev	AAAAACTCGAGTTATTTATATTGTGGGATGTCG	*Xho*I
mprF_for	ATTGGATCCTTTCAATGTTGACAGGTTTAA	*Bam*HI
mprF_rev	ATTGAATTCGACGTAACATCTTTAGCAGG	*Eco*RI

References

1. Hsieh MH, Yu CM, Yu VL, Chow JW. 1993. Synergy assessed by checkerboard. A critical analysis. Diagn Microbiol Infect Dis 16:343–349. doi:10.1016/0732-8893(93)90087-n.
2. Peschel A, Otto M, Jack RW, Kalbacher H, Jung G, Götz F. 1999. Inactivation of the dlt operon in Staphylococcus aureus confers sensitivity to defensins, protegrins, and other antimicrobial peptides. J Biol Chem 274:8405–8410. doi:10.1074/jbc.274.13.8405.
3. Berscheid A, François P, Strittmatter A, Gottschalk G, Schrenzel J, Sass P, Bierbaum G. Generation of a vancomycin-intermediate Staphylococcus aureus (VISA) strain by two amino acid exchanges in VraS. J Antimicrob Chemother. 2014 69:3190-8. doi: 10.1093/jac/dku297.
